# A rare cecal subepithelial tumor in a Crohn´s Disease patient

**DOI:** 10.4322/acr.2020.211

**Published:** 2020-12-14

**Authors:** Joana Inês Alves da Silva, Cidalina Caetano, Anabela Maria Sousa da Rocha, Nuno Jorge Lamas, Paula Lago, Isabel Maria Teixeira de Carvalho Pedroto

**Affiliations:** 1 Centro Hospitalar Universitário do Porto, Department of Gastroenterology, Porto, Portugal; 2 Centro Hospitalar Universitário do Porto, General Surgery Service, Colorectal Unit, Porto, Portugal; 3 Centro Hospitalar Universitário do Porto, Pathology Department, Anatomic Pathology Service, Porto, Portugal; 4 University of Minho, School of Medicine, Life and Health Sciences Research Institute (ICVS), Braga, Portugal; 5 ICVS/3B’s, PT Government Associate Laboratory, Braga, Guimarães, Portugal; 6 University of Porto, Abel Salazar Biomedical Sciences Institute, Porto, Portugal

**Keywords:** Appendiceal tumors, Crohn´s Disease, Neoplasms, Cystic, Mucinous, and Serous

## Abstract

Appendiceal tumors comprise a variety of histologic types, including appendiceal mucinous neoplasms, which can be grouped as premalignant lesions, tumors of uncertain malignant potential, and malignant lesions. The appendiceal mucinous neoplasms are characterized by mucinous epithelial proliferation with extracellular mucin and pushing tumor margins, commonly an incidental finding during operative exploration. We report the case of a low-grade appendiceal mucinous neoplasm presenting as a subepithelial lesion in Crohn´s Disease patient. The diagnosis was not straightforward, and only surgical resection allowed an accurate diagnosis. Although Inflammatory Bowel Disease is a risk factor for the development of colorectal neoplasms, the absolute risk for appendiceal tumors is uncertain. The frequency of progression to malignancy remains to be determined.

## INTRODUCTION

Appendiceal tumors, accounting for less than 1% of all cancers, combine an array of histologic sub-types, including appendiceal mucinous neoplasms (AMN). The malignant potential of these lesions is variable, and the clinical course appears to be determined by the stage at diagnosis and its histological features. Well-differentiated mucin-producing appendiceal tumors have a better prognosis. Early-stage AMNs most commonly appear as incidentalomas after resection for suspected appendicitis, comprising approximately 1% of appendectomy specimens. Advanced stage disease may present with abdominal distension due to the accumulation of mucin in the peritoneal space, which may occur in about 20% of patients with a mucinous neoplasm of appendix.^1^ ^,^ ^2^

Low-grade appendiceal mucinous neoplasms (LAMN) are the most frequent sub-group of AMNs, which are characterized by mucinous epithelial proliferation with extracellular mucin and pushing tumor margins.^3^ The distinctive feature of LAMN is the low-grade cytologic atypia combined with the absence of signs that indicate invasive infiltration of the appendiceal wall.^4^ The disruption of the normal appendiceal mucosal architecture, even if focal, with loss of the lamina propria and muscularis mucosa, as well as, fibrosis, hyalinization and calcification of the appendiceal wall with lymphoid follicle atrophy in the submucosa are typically observed in LAMN.^3^ ^,^ ^4^ Different degrees of extracellular mucin can be observed, without extension into the sub-serosa.^5^ LAMNs account for nearly 1% of all colorectal malignancies, with the peak incidence in the sixth decade of life. There is equal frequency among men and women.^3^ ^,^ ^6^ The most feared complication is the extravasation and seeding of mucin and neoplastic cells into the adjacent peritoneum, leading to pseudomyxoma peritonei, which is associated with a high mortality rate and occurs in late stages of the disease.^2^

Inflammatory Bowel Disease (IBD) is a risk factor for colorectal cancer development. Although IBD usually occurs with microscopic involvement of the appendix, its contribution to the development of appendiceal neoplasms remains to be determined.^7^ ^,^ ^8^

Here, we report a case of a LAMN presenting as a subepithelial tumor in a patient diagnosed with Crohn´s Disease (CD), comprehensively review the existing literature on the subject and discuss the important implications on the follow-up of patients diagnosed with IBD arising from the possible association between both entities.

## CASE REPORT

We report the case of a 50-year-old woman, who was diagnosed with Crohn’s Disease at 38-years-old. According to the Montreal Classification of IBD, the patient is classified as A3 L2+ L4B1 due to her age and the involvement of the esophagus, ileum, colon, and anorectal region. Continuous azathioprine therapy led to optimal disease control. For several years, the patient was in clinical and analytical remission with only mild endoscopic activity.

At 49-years old, a follow-up ileocolonoscopy revealed a 2cm subepithelial lesion in the cecum’s appendicular area. The histological analysis of the biopsied material showed mild inflammation. In addition, discrete cicatricial findings in all colon were reported, and several pseudo-polyps in the transverse colon were removed.

**Figure 1:**
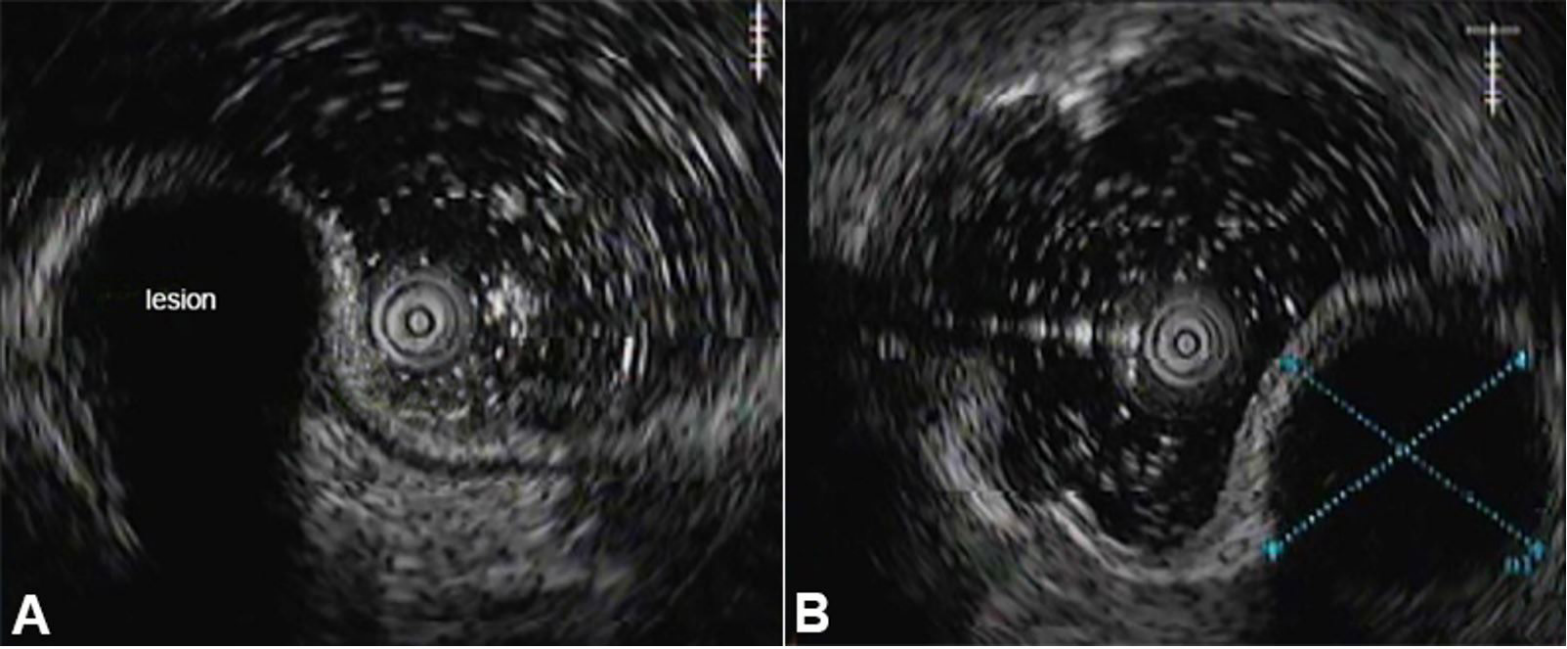
Endoscopic ultrasound of the cecum. Intramural lesion measuring in the cecum’s appendicular orifice. The dependence of the lesion on the submucous (**A**) or muscularis propria (**B**) layers was not securely ascertained.

To better characterize the subepithelial lesion, the patient underwent endoscopic ultrasound (EUS) (Figure 1), which demonstrated an intramural lesion measuring 19,1 x 18mm in the cecum’s appendicular orifice, displaying regular and well-defined contours, homogenous and hypoechoic.

The dependence of the lesion on the submucous or muscularis propria layers was not securely ascertained, which implied distinct differential diagnosis. Regional adenopathy, in the colon, were not identified.

She was then submitted to computerized tomography (CT) enterography, which did not reveal mucosal thickening of the cecum or enhancement patterns.

After a case discussion in the Inflammatory Bowel Disease Multidisciplinary Group Meeting, the patient underwent laparoscopic ileocecal resection.

**Figure 2:**
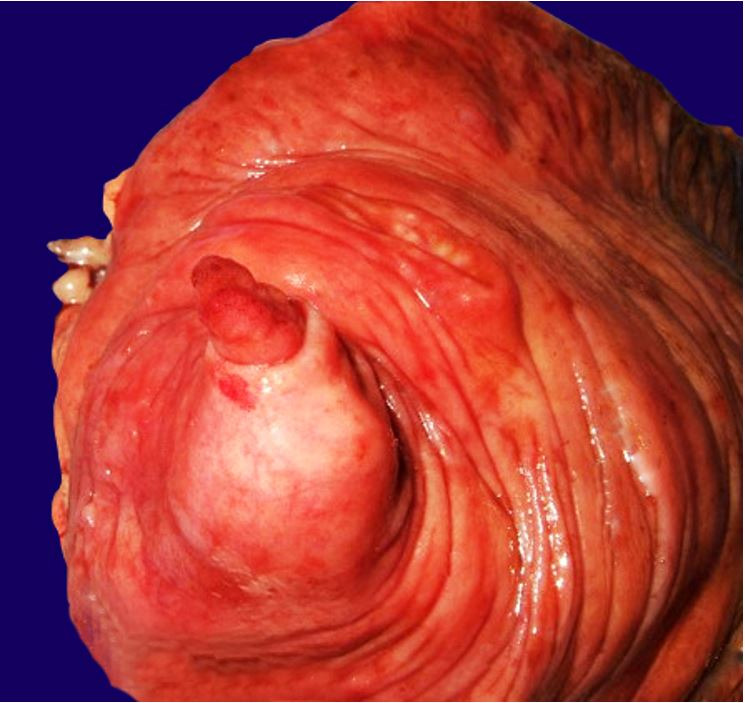
Macroscopic aspects of the operative specimen: appendicular tumor and appendicular mucocele.

Surgically resected specimen comprised a 13cm terminal ileum segment, a 6cm cecocolic segment, and a 3,5cm ileocecal appendix (shown in Figure 2).

**Figure 3:**
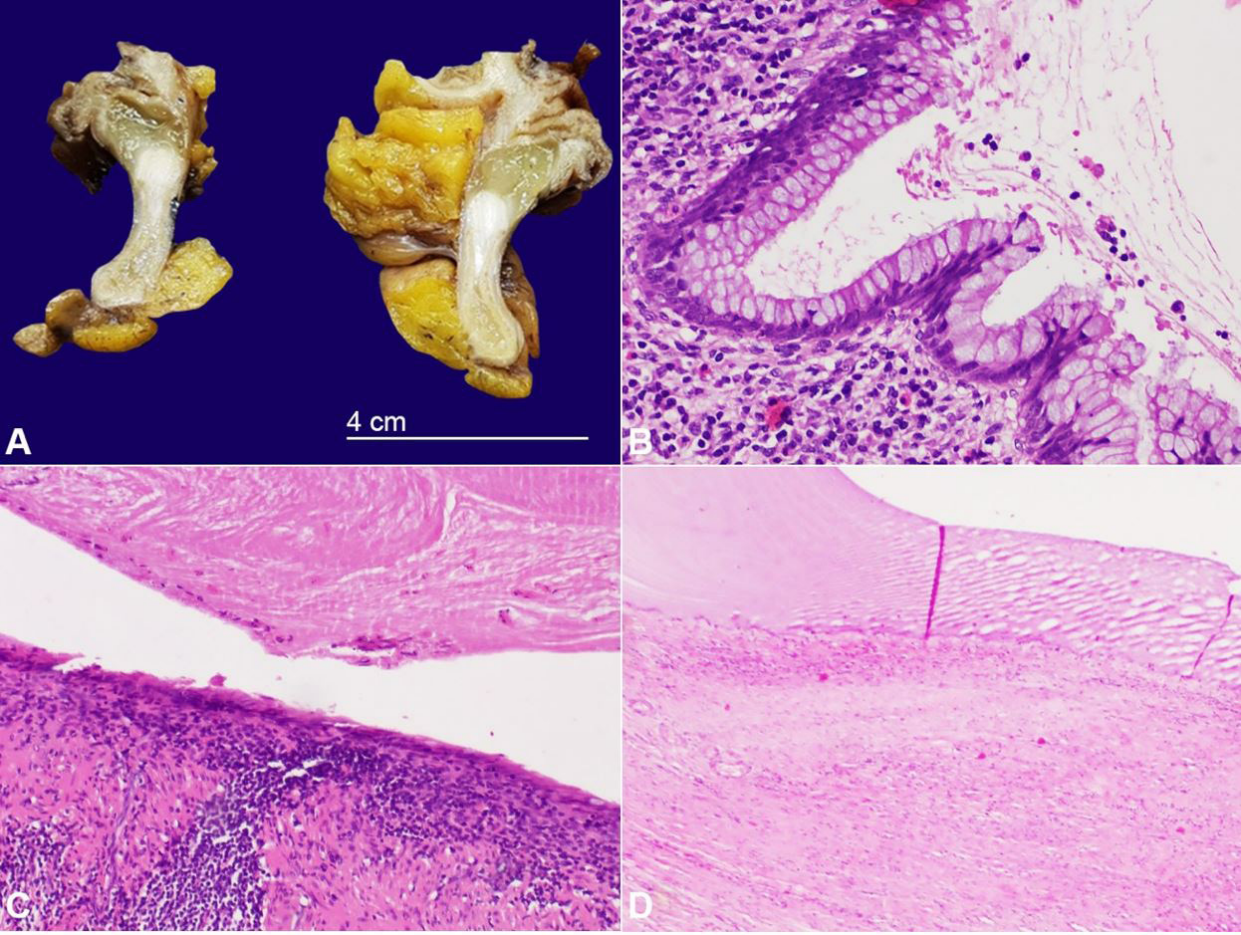
**A** – Macroscopic view of the ileocecal appendix removed from the surgical specimen. The lumen is distended due to the accumulation of mucinous material that appears to be confined to the appendix; **B** – An area of preserved epithelium of the appendiceal mucinous neoplasm, which has proliferated mucinous epithelium with tall cytoplasmic mucin vacuoles and compressed bland nuclei, focally with a vaguely undulating appearance (H&E, 200x); **C** – Marked expansion of the appendiceal lumen by mucin, leading to the disruption of the normal architecture of the mucosa and submucosa, which has decreased amount of lymphoid tissue and mild fibrosis (H&E, 200x); **D** – Marked distention of the appendiceal lumen by mucin, leading to the destruction of the mucosa and submucosa. The abundant mucinous material is contained by the muscular propria layer and was not shown to involve the sub-serosa (H&E, 200x).

No relevant histological findings were observed in the ileum and colon. However, the ileocecal appendix had a smooth serosa, irregular caliber, and a dilated lumen filled with abundant mucinous content and a whitish nodular area measuring 1,1x1x0,7cm in the middle third. The appendix was entirely submitted for histologic evaluation, and a diagnosis of LAMN was made (Figure 3). Acellular mucin was confined to the muscular propria of the appendix without involvement of the sub-serosa. There was no evidence of lymph node and perineural involvement. The surgical margins were uninvolved by the neoplasm. The neoplasm was staged as pTis(LAMN) N0 R0 [American Joint Committee on Cancer (AJCC) Cancer Staging System, 8th Edition, 2017].^9^

## DISCUSSION

The available literature on the importance of colonoscopy for AMN diagnosis is scarce, but colonoscopy may reveal a pathognomonic “volcano sign”, so-called to describe an encroaching mass which obstructs the appendiceal opening with a central crater that produces mucin.^10^ Additional imaging modalities for the diagnosis include EUS and CT scan. In our case, the patient did not have any typical characteristic feature in a colonoscopy or CT scan. The definitive diagnosis was made only after elective surgery.

LAMN significantly distorts the appendix architecture, making it challenging to stage using the conventional criteria. The eighth edition of the American Joint Committee on Cancer (AJCC) created a new T category specifically for LAMN, termed the Tis(LAMN) with prognostically relevant criteria.^9^ Tis(LAMN) refers to a LAMN confined to the muscularis propria, and patients with this stage have minimal risk for recurrent disease after complete resection.^9^ In our case, there was no pathological evidence of malignancy involvement beyond the appendiceal muscularis propria, or lymph node metastasis and no evidence of malignant cells in the mucin pools in the peri-appendiceal tissue. Thus, no further surgical or adjuvant therapies were required. Adjuvant chemotherapy is not recommended for low-grade well-differentiated mucinous tumors, although it may be considered in specific situations where cancer shows invasive features.^2^

Most literature data suggest that simple appendectomy is satisfactory for tumors exhibiting only local disease. Right hemicolectomy should be considered to clear the tumoral margin in cases of histological evidence of colic involvement after an appendectomy, and it should also be considered for tumors involving the peri-appendiceal area, tumors larger than 2 cm, high-grade histology or tumors that invade the muscularis propria.^2^ ^,^ ^11^ Recently, there has been an increase in laparoscopic surgery for LAMNs, as this approach is minimally invasive, with minimal postoperative pain and faster recovery. Despite this, when selecting a laparoscopic versus open procedure, careful consideration must be given to minimize rupture and mucinous seeding.^6^ ^,^ ^10^

Although there is an established association between gastrointestinal cancer and CD, the association between appendiceal mucinous neoplasms and CD is not well established. Previous studies have reported that patients with IBD have increased risk of developing colorectal carcinoma approximately one decade after disease onset. Although appendiceal inflammation occurs histologically in 40%–86% of colectomy specimens from patients with IBD,^12^ appendiceal neoplasms are not frequent.^13^ ^,^ ^14^

On a comprehensive literature review, we found only 6 studies on concomitant IBD and AMN, comprising four reports in CD and eleven in ulcerative colitis^7^ ^,^ ^15^ ^-^ ^19^ which are summarized in Table 1.

**Table 1:** AMN in Patients with IBD

#	Reference	Age/ Gender	IBD type	Disease extent	Presentation	Treatment
1	Hernández Benabe et al.^15^	16/M	CD	Ileitis	Acute peritonitis	Emergency laparoscopic ileocecectomy with primary ileocolonic anastomosis
2	Wong and Darwin^16^	62/F	UC	In remission	Incidental finding of at the appendiceal orifice during surveillance colonoscopy.	Laparoscopic appendectomy
3.		34/F	UC	Pancolitis	Incidental finding of a bulging appendiceal orifice	Appendectomy
4.	Lakatos et al.^18^	54/M	UC	Proctosigmoiditis	Abdominal pain	Ileocecal resection
5.	Orta et al.^7^	76/F	UC	Left-sided	-	-
6.		24/M	UC	Pancolitis	-	-
7.		34/M	UC	Pancolitis	-	-
8.		57/F	UC	Left-sided	-	-
9.		28/F	UC	Pancolitis	-	-
10.		75/M	UC	Pancolitis	-	-
11.		43/M	CD	Ileocolitis	-	-
12.		43/M	CD	Ileocolitis	-	-
13.		36/M	CD	Ileicolitis	-	-
14.		65/F	UC	Unknown	-	-
15.	Lyda et al.^19^	48/M	UC	Pancolitis	Concomitant diagnosis of AMN and four invasive adenocarcinomas	Ileocolectomy

AMN, appendiceal mucinous neoplasms; CD, Crohn´s Disease; F, female; IBD, inflammatory bowel disease; M, male; UC, ulcerative colitis.

Lumarg Orta et al.^7^ performed a retrospective study on incidental appendiceal neoplasms in colectomy specimens of adults with and without IBD showing that appendiceal cystadenomas, now called AMNs, were 15-fold more prevalent among IBD patients with synchronous colorectal neoplasia compared with controls.

Disease duration and histological inflammatory activity have been directly implicated as independent risk factors for the development of colorectal dysplasia and carcinoma in patients with IBD.^7^ ^,^ ^8^ As such, it might be hypothesized that appendiceal inflammation related to IBD may predispose to analogous forms of appendiceal neoplasia. The rarity of these neoplasms may reflect the small area of mucosa at relative risk to that of the colorectum, as well as the inherent diagnostic challenges.

The possible recognition of AMNs as another neoplastic complication of IBD strengthens the need for clinical awareness when these patients undergo screening for colorectal cancer.
